# Postpartum Uterine Involution in Cows: Quantitative Assessment of Structural Remodeling and Immune Cell Infiltration

**DOI:** 10.3390/ani15172520

**Published:** 2025-08-27

**Authors:** Karine V. Aires, Ana Paula da Silva, Leonardo G. de Andrade, Alexandre Boyer, Gustavo Zamberlam, Valerio M. Portela, Alfredo Q. Antoniazzi, Guillaume St-Jean

**Affiliations:** 1 Laboratory of Biotechnology and Animal Reproduction (BioRep), Federal University of Santa Maria, Santa Maria 97105-900, RS, Brazil; 2 Centre de Recherche en Reproduction et Fertilité, Université de Montréal, Saint-Hyacinthe, QC J2S 2M2, Canada

**Keywords:** endometrium, inflammation, postpartum, uterine biopsy, uterine involution

## Abstract

Postpartum uterine involution in cattle involves complex morphological and immunological changes essential for restoring uterine health and fertility. This study evaluated endometrial biopsies collected at four postpartum time points to characterize tissue remodeling and immune cell dynamics during involution. Histology revealed intact luminal columnar epithelium in 92.98% of samples, with stable stromal architecture. Stromal edema decreased by Day 7 but increased again by Day 35, while endometrial gland numbers significantly rose at Day 35, suggesting glandular recovery linked to resumed cyclicity. Subepithelial collagen deposition peaked on Day 21, indicating active extracellular matrix remodeling. Immunologically, early postpartum was marked by increased PMNs and macrophages, whereas Day 21 showed peak infiltration of natural killer (NK) cells and T and B lymphocytes, sometimes forming lymphoid aggregates. Manual and automated immune cell quantifications correlated well. These findings demonstrate a dynamic shift from acute neutrophil-dominated inflammation to a lymphocyte-rich environment during uterine involution. This immune modulation may contribute to the earlier diagnosis of subclinical endometritis, typically identified at later stages of postpartum period. Overall, this study provides insight into the temporal immunomorphological events supporting uterine recovery, with potential implications for reproductive management in dairy cattle.

## Simple Summary

After giving birth, dairy cows go through a recovery process called uterine involution, which is essential for them to become fertile again. During this period, the uterus must heal and return to its normal state. In this study, we examined samples of the uterine lining (endometrium) from cows at different times after calving to understand how the tissue changes and how immune cells behave during recovery. We found that the uterine tissue remained generally well-structured, and by 35 days after calving, there was a noticeable increase in uterine endometrial glands, suggesting that the cows were resuming their reproductive cycles. Inflammation in the uterus changed over time: right after calving, there were more neutrophils and macrophages—types of immune cells that fight infection. By three weeks postpartum, other immune cells such as natural killer (NK) cells and lymphocytes became more common. These immune alterations may represent potential early biomarkers for the detection of uterine diseases, like subclinical endometritis. Our findings help improve the understanding of how the cow’s uterus recovers after parturition and may contribute to better reproductive care in dairy farming.

## 1. Introduction

Calving causes mechanical injury to the epithelial and underlying uterine tissues due to placental separation. The subsequent recovery is influenced by the severity of tissue trauma, metabolic stress, and immune competence during the transition period [1,2,3]. The establishment of pregnancy in ruminants depends on key morphological and functional adaptations in the endometrium. These involve cyclic regulation of epithelial and stromal cell proliferation and apoptosis [4]. Following parturition, uterine involution occurs to restore the uterus to a state capable of supporting a new pregnancy within 6 to 8 weeks [5]. This finely regulated process encompasses macroscopic, microscopic, and molecular changes, including uterine contractions, tissue atrophy, necrosis, sloughing, and endometrial regeneration. The most substantial uterine size reduction occurs between 10 and 14 days postpartum in cows undergoing physiological puerperium [6].

Studies on uterine health, involution, and maternal–embryonic communication often rely on clinical, hormonal, or histopathological data. Among these, endometrial biopsy is considered a safe and representative technique for evaluating uterine health and integrity [7,8]. Histological and morphological analysis of endometrial biopsies can help identify infertility-related changes and assess inflammatory status and concurrent tissue lesions [8,9].

Histopathological and molecular profiling of the early postpartum uterus reveals a characteristically inflammatory environment [6]. This is relevant to understanding the pathogenesis of endometritis, a prevalent postpartum disease with significant impact on reproductive performance and herd economics. Endometritis ranges from acute to chronic uterine inflammation, often arising from bacterial contamination and exacerbated by dystocia, retained placenta, or metabolic stress [10]. Persistent pathogenic bacteria or dysregulated immune responses may lead to uterine disease or dysbiosis [11,12]. More than half of all dairy cows are affected to some extent, with consequences including impaired ovarian and endometrial function [13].

Subclinical endometritis, often diagnosed cytologically, may go undetected, allowing chronic inflammation and irreversible lesions such as fibrosis to develop, thus compromising fertility [13,14,15,16]. Histologically, the condition is marked by epithelial disruption, infiltration of neutrophils, macrophages, eosinophils, and mast cells, along with vascular congestion, stromal edema, and lymphoplasmacytic accumulation [14]. Disease progression is influenced by both the virulence of pathogens and the animal’s immune tolerance and resistance, which are shaped by genetic and environmental factors. However, peripartum stressors such as dystocia, parity, and ketosis often outweigh genetic predisposition as predictors of disease [2].

To better understand endometritis pathogenesis and support prevention and early diagnosis, it is essential to deepen our knowledge of physiological uterine involution and the local inflammatory responses that distinguish health from disease. Healthy involution is characterized by controlled tissue remodeling and a quantifiable influx of inflammatory cells in the endometrium. Mapping these responses may help clarify the boundary between physiological and pathological inflammation and support the early identification of cows at risk for endometritis.

Given the limited temporal resolution of prior studies, we hypothesize that immune cell dynamics during involution follow a regulated timeline that may be critical for uterine recovery and fertility. The objective of this study was to characterize the morphological changes and physiological inflammatory responses occurring during normal postpartum uterine involution in cows, as well as to quantify the inflammatory cell populations present in the healthy endometrium. Together, these analyses aim to improve our understanding of the key stages of uterine involution and to identify features potentially associated with the transition to pathological conditions.

## 2. Materials and Methods

### 2.1. Animals

Multiparous Holstein cows (*Bos taurus taurus*; *n* = 59; in their second or later lactation) in good health and without evidence of endometrial disease were selected at various stages of postpartum uterine involution from seven commercial dairy farms located in the northwest region of Rio Grande do Sul, Brazil. Animals were selected based on their parturition date, and the absence of dystocia or problems reported during labor. Before collecting the samples, a complete physical exam was perform to classify as clinically healthy based on specific clinical, reproductive, and productive criteria: absence of clinical conditions such as endometritis, metritis, mastitis, milk fever, or retained placenta; normal rectal temperature (38.0 °C to 39.3 °C); absence of purulent uterine discharge beyond 21 days postpartum; absence of subclinical endometritis on day 35 through cytobrush analysis, and an average body condition score (BCS) of 3 +/− 0.25 on a 1–5 scale. All health assessments and sample collections were performed by the same veterinarian on seven different client farms during regular herd visits. When possible, samples from the different farms were distributed proportionally in each group based on the size of the herd. The study protocol was reviewed and approved by the Animal Ethics Committee of the Federal University of Santa Maria (CEUA/UFSM; protocol number 060654).

### 2.2. Uterine Biopsies and Experimental Design

Endometrial biopsies were collected from cows (*n* = 59) using a Hauptner endometrial biopsy forceps (Solingen, Germany), following the technique described by Chapwanya et al. [7]. After cleaning the perineum and external genitalia, the biopsy instrument, enclosed in a protective sheath, was introduced into the vagina. The instrument was guided through the cervix via rectal manipulation. Following rupture of the sheath at the external cervical os, biopsy forceps were introduced into the uterus and carefully directed as medially as possible, especially on Days 2 and 7 in the previously gravid uterine horn. Only the samples collected in the intercaruncular regions were evaluated (to ensure consistency throughout the analyses). To ensure consistent tissue sampling, the tip of the instrument was located rectally. The jaws of the forceps were then opened, and the medial uterine wall was gently pressed against the open jaws using the hand in the rectum. Endometrial tissue was obtained by closing the jaws and withdrawing the instrument [7]. All procedures were performed by the same veterinarian to ensure consistency. A minimum of 10 samples per group was targeted based on previously cited literature and the availability of farms and owner’s consent [6,17]. Biopsies were collected from different cows (*n =* 59) at four postpartum time points: Day 2 (*n* = 11), Day 7 (*n* = 15), Day 21 (*n* = 13), and Day 35 (*n* = 20). All samples were obtained from the as medially portion as possible in the previously gravid uterine horn, as limitations were encountered by the size of uterus on Days 2 and 7 postpartum. Immediately after collection, biopsies were placed in conical tubes containing 10 mL of 10% buffered formalin.

### 2.3. Cytology Evaluation

Cows in the Day 35 group underwent cytological evaluation for the diagnosis of subclinical endometritis. Endometrial epithelial and polymorphonuclear (PMN) cells were collected using a cytology brush (Kolplast Group, São Paulo, Brazil) before endometrial biopsy collection. The cytobrush was attached to the tip of a standard artificial insemination (AI) applicator, which was covered with a disposable AI sheath and protected by a sanitary sheath, following previously established protocols [18,19]. The device was introduced into the uterus via the cervix, and the brush was rotated to collect cells from the uterine body. The cytobrush was then detached, and samples were prepared by gently rolling the brush onto microscope slides. Slides were stained using a commercial staining kit (Quick Panoptic^®^, Laborclin, Pinhais, Brazil) as described by Krause et al., (2014) [20]. Each slide was examined under 400× magnification by two independent evaluators. A total of 200 cells per slide—including PMNs, mononuclear cells, and epithelial cells—were counted to determine the proportion of PMNs in the sample [18]. Subclinical endometritis was diagnosed when the proportion of PMNs exceeded 6% [21]. Animals diagnosed with subclinical endometritis (*n =* 9) were excluded from the study.

### 2.4. Histopathology Evaluation of Uterine Morphology

Endometrial biopsies were collected, fixed in 10% neutral buffered formalin, embedded in paraffin, sectioned at 3 µm thickness, and stained with hematoxylin and eosin (HE) or hematoxylin–eosin–phloxine–saffron (HePS). Stained sections were scanned at 400× magnification using the Aperio ScanScope CS whole-slide scanner (Leica Biosystems, Wetzlar, Germany). The integrity of the luminal epithelium and underlying stroma was assessed subjectively, while additional histopathological features—including vascular congestion, stromal hemorrhage, and edema—were evaluated semi-quantitatively using a scoring system ranging from 0 to 4 (0 = absent, 1 = minimal, 2 = mild to moderate, 3 = marked, and 4 = severe), under light microscopy. Criteria used for morphological assessment are summarized in Table 1. The number and appearance of endometrial glands (see Supplementary Figure S1) was counted in each section. Straight endometrial glands correspond to elongated glands (Figure S1C,D) whereas coiling refer to a collection of regrouped glands visible in transverse section (due to the coiling) (Figure S1A,B). Dilation corresponds to increased size of the endometrial gland visible lumen due to dilatation (Figure S1B,D). Fibrosis is evaluated by the number of layers of fibroblasts around the glands. The inflammation evaluated corresponded to the type and number of inflammatory cells infiltrating the structures and/or the surrounding stroma.

For PMN quantification, ten high-power fields (HPFs; 400× magnification) were randomly selected across the epithelium and stratum compactum, and the average number of PMNs was calculated as previously described [22]. All histological evaluations were performed by a single blind examiner.

### 2.5. Immunohistochemical Evaluation of Uterine Immune Cells During the Postpartum Period

Tissue sections were deparaffinized in toluene and rehydrated through a graded ethanol series. Heat-mediated antigen retrieval was performed using sodium citrate buffer. Endogenous peroxidase activity was blocked by incubation in 3% hydrogen peroxide for 10 min. Primary antibodies (targeting inflammatory cell markers; Table 2) were diluted in TBST containing 5% goat serum and incubated overnight at 4 °C. Negative control sections were processed in parallel without the primary antibody.

Immunodetection was performed using the Vectastain^®^ Elite ABC HRP Kit (Vector Laboratories; Newark, CA, USA, PK-6101) and the DAB substrate kit (3,3′-diaminobenzidine; Vector Laboratories; Newark, CA, USA, SK-4100), according to the manufacturer’s instructions. Slides were counterstained with hematoxylin and mounted for analysis. Photomicrographs were obtained either using a light microscope at 400× magnification (Carl Zeiss, Oberkochen, Germany) and scanned at the same magnification using the Aperio ScanScope CS system (Leica Biosystems).

Quantitative immunohistochemical analysis was conducted using the Aperio ImageScope software (version 12.4.6, Leica Biosystems). A total of six 400× high-power fields (HPFs), distributed between the luminal epithelium and the stratum compactum of each biopsy, were manually annotated. Positive cells (e.g., PMNs) were identified and individually counted within each annotated HPF. The average number of positive cells per HPF was calculated for each sample and used for subsequent statistical analyses. All evaluations were performed by a single examiner blinded to sample identity and group allocation.

### 2.6. Quantitative Analysis with QuPath© 0.4.3 Software

Stained biopsy sections were scanned at 400× magnification using the Aperio ScanScope CS whole-slide scanner (Leica Biosystems). The resulting digital images were imported into QuPath© (version 0.4.3), where they were reviewed during the scoring process. Sections exhibiting artifacts that interfered with analysis—such as tissue folding—were manually excluded.

To correlate the manual quantitative method employed in this study with an automated image analysis approach, QuPath© detection parameters were individually optimized for each antibody using the Optical Density Sum (ODS) detection algorithm. For automated cell detection, six annotation areas per biopsy (one per square object; total of six per animal, *n =* 10 animals per group) were created using elliptical shapes and placed at 400× magnification. These annotated areas corresponded to six high-power fields (HPFs) per sample and were randomly distributed between the luminal epithelium and the stratum compactum to ensure maximal tissue representativeness (see Supplementary Figure S2). The number of positively stained cells and the corresponding detected area were used to calculate the average number of positive cells per mm^2^. These results, along with markup images indicating the identified cells, were exported for visual verification [23]. The quantitative data were tabulated, and the mean number of positive cells per mm^2^ was subsequently calculated for each group.

### 2.7. Statistical Analyses

Statistical analyses were performed using JMP software, version 7.0 (SAS Institute Inc., Cary, NC, USA). The nonparametric Wilcoxon/Kruskal–Wallis’s test was used to evaluate differences in immune cell infiltration between experimental groups and to analyze semiquantitative histological scores. When appropriate, differences between group means were assessed using the Tukey–Kramer Honestly Significant Difference (HSD) test. Homogeneity of variances was tested using O’Brien’s test.

To assess the correlation between manual and automated cell quantification methods, Spearman’s rank correlation coefficient was calculated. Data are presented as mean ± standard error of the mean (S.E.M.), and statistical significance was set at *p* < 0.05. Agreement between manual and automated quantification of positively marked cells in the bovine endometrium was assessed using Bland–Altman plots. For each sample, the mean of the two measurements was plotted on the *X*-axis, and the difference between them (manual − automated) on the *Y*-axis. The bias (mean difference) was calculated as the average of all differences, and the limits of agreement (LoA) were defined as bias ± 1.96 × standard deviation of the differences. The presence of proportional bias was evaluated by regression analysis of the differences against the means. Bland–Altman plots were generated in GraphPad Prism version 9.0 (GraphPad Software, San Diego, CA, USA) using descriptive statistics calculated in JMP version 7 (SAS Institute, Cary, NC, USA). Numerical values for the bias and LoA were reported in the plots.

## 3. Results

### 3.1. Overall Morphological Characterization of the Uterus During Postpartum Involution

**Figure 1:**
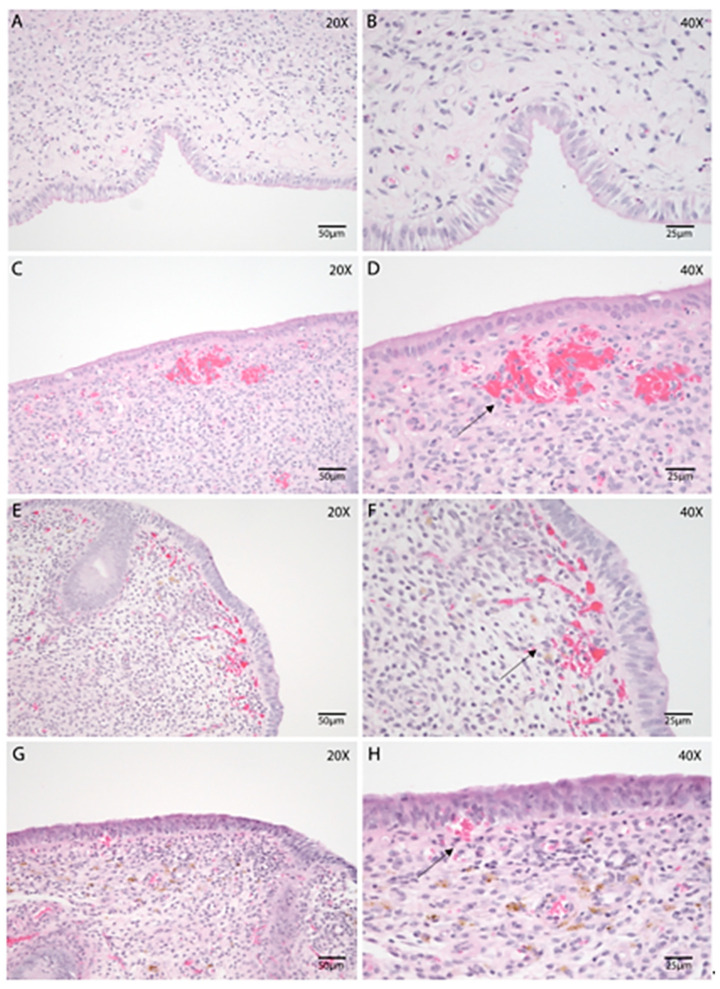
Morphological evaluation of the bovine endometrium throughout the postpartum involution using endometrial biopsies. Hematoxylin and Eosin (HE). (**A**–**H**) Endometrial biopsies of bovine uterus on Days 2 (**A**,**B**), 7 (**C**,**D**), 21 (**E**,**F**), and 35 postpartum (**G**,**H**). B, D, F, and H represent higher magnification of A, C, E, and G, respectively. (**A**,**B**) Mild stromal edema and variable PMN infiltration are present in the stroma. (**C**,**D**) Epithelial vacuolation with mild subepithelial edema, collagen deposition and, abundant blood vessels and hemorrhages (black arrow) can be noted on Day 7 postpartum. (**E**,**F**) Subepithelial hemorrhage (black arrow) and stromal edema are often noticeable on Day 21. (**G**,**H**) A few hemorrhages (black arrow) are noted in the SC, along with edema, on Day 35. Images were obtained using Aperio ImageScope x64 12.4.6 software (Leica Biosystems), left column magnification 200×, scale 50 µm, right column magnification 400×, scale 25 µm.

Uterine biopsies collected during the postpartum period were subjected to morphological assessment to evaluate structural characteristics and adaptations associated with uterine involution. An intact luminal epithelium (Figure 1A,B) was identified in 92.98% of the samples, all of which exhibited a columnar epithelial phenotype. When present, the epithelium appeared morphologically preserved, with a height ranging from 15 µm to 30 µm. Infiltration of both mononuclear and polymorphonuclear (PMN) inflammatory cells was observed within the epithelial layer of all samples, often accompanied by subepithelial edema (Figure 1C,D). Collection-related artifacts, such as epithelial desquamation or focal hemorrhage, were occasionally present (Figure 1H).

The structural organization of the stratum compactum and stratum spongiosum (Figure 1) remained relatively consistent across the different stages of uterine involution. Both layers displayed evidence of stromal inflammation and contained endometrial glands. Vascular dilation and congestion were frequently noted in the stratum compactum (Figure 1C,D). As expected, endometrial glands were more prominent in the stratum spongiosum (Figure 1E,G).

### 3.2. Quantitative and Semiquantitative Evaluation of Morphological Changes During the Postpartum Period

**Figure 2:**
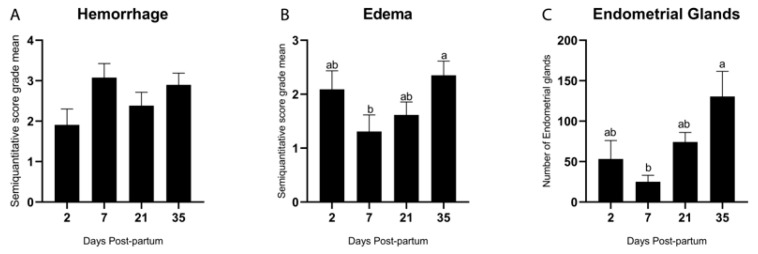
Semiquantitative evaluation (score of 0 to 4) of hemorrhage (**A**) and edema (**B**) and quantitative evaluation of the endometrial glands in the bovine endometrial stroma during the postpartum period (**C**). Different letters are statistically significant (*p* < 0.05). The absence of letters denotes no significant difference among groups. Results are presented as mean ± standard error of the mean (S.E.M.).

A semiquantitative evaluation of hemorrhage (Figure 2A) and stromal edema (Figure 2B) within the stratum compactum and stratum spongiosum was performed across different postpartum time points. Both alterations were observed throughout the involution process and were occasionally associated with the presence of hemosiderin (Figure 1H). The severity of hemorrhagic changes remained relatively constant, with no statistically significant differences among time points (*p* = 0.21; Tukey–Kramer HSD test) (Figure 2A). In contrast, the presence of stromal edema (Figure 2B) decreased significantly by Day 7 (*p* = 0.01) and increased again on Day 35 (*p* = 0.01; Tukey–Kramer HSD test).

As described by Spencer et al. [24], endometrial glands originate from the luminal epithelium and develop into a network of coiled tubules extending through the stroma to the myometrium. Endometrial glands (Figure 2C) were quantitatively assessed, and a significant increase in the number of glands was observed on Day 35 postpartum (*p* = 0.002; Tukey–Kramer HSD test). A detailed characterization of the glands was performed based on previously established parameters [25], including their density, morphological type, size, and the presence of peri glandular or intraglandular inflammation, as summarized in Table 3. The morphological classification of endometrial glands as straight, coiled, and dilated across postpartum Days 2, 7, 21, and 35 provides insights into the progression of endometrial regeneration in cattle. Early predominance of straight glands likely reflects limited morphogenesis, while later appearance of coiled and dilated forms indicates advancing adenogenesis and secretory recovery. For reference to the details evaluated in Table 3, please refer to Table 1.

**Figure 3:**
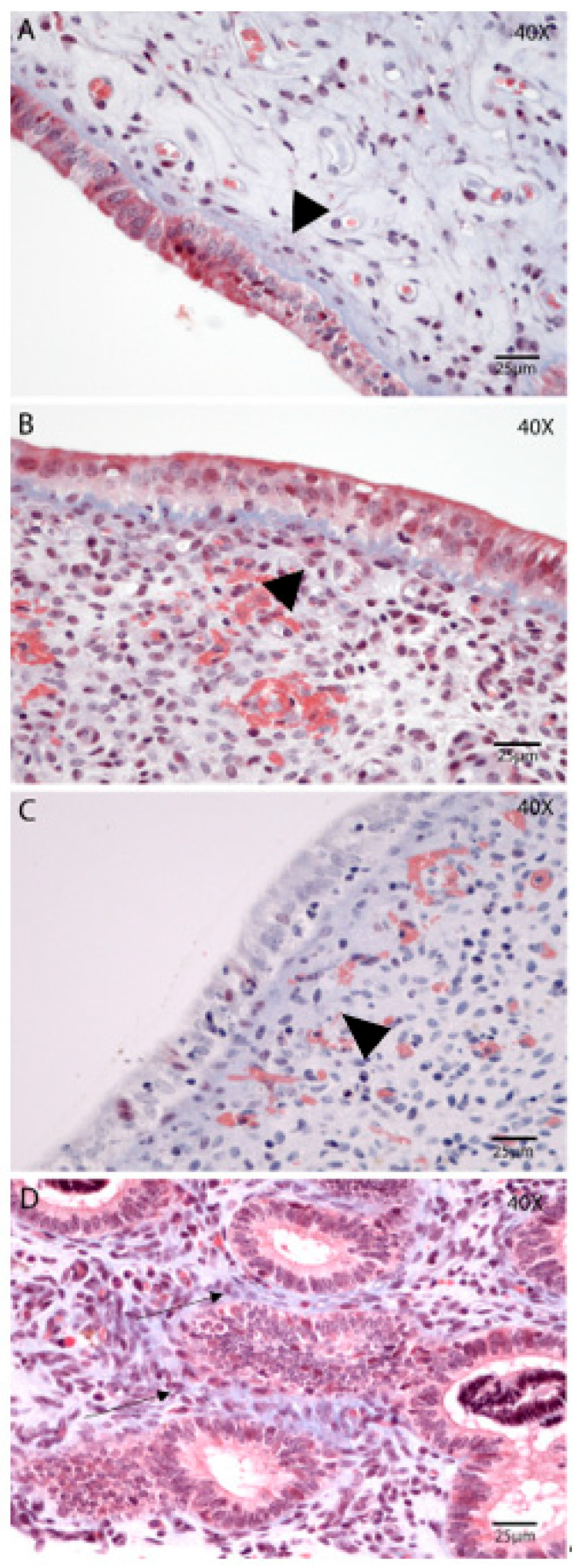
Extracellular matrix reorganization (collagen) during postpartum uterine involution. Masson’s Trichrome. Endometrial biopsies of bovine uterus on Days 2 (**A**), 7 (**B**), 21 (**C**), and 35 postpartum (**D**). (**A**) The SC is readily visible, with compacted collagen deposition, on Day 2 postpartum (arrowhead). (**B**,**C**) There is a tendency for increased thickness of the SC on Day 7 and Day 21 postpartum (arrowhead). (**D**) Day 35 postpartum, the glands are more abundant in the SS, along with increased peri glandular deposition of collagen (black arrow). The images were obtained using Aperio ImageScope x64 12.4.6 software (Leica Biosystems), magnification 400×, scale 25 µm.

The distribution of collagen fibers and fibrotic areas in the endometrium was assessed using Masson’s Trichrome staining (Figure 3A–D). Collagen depositions were identified as a normal feature of the involution process. On Day 2 postpartum (Figure 3A), collagen fibers were evident in all uterine compartments and showed a tendency to decrease as involution progressed. Notably, on Day 21 (Figure 3C), there was a significantly higher incidence of subepithelial collagen deposition in the stratum compactum compared to other time points (*p* = 0.01; Tukey–Kramer HSD test). On Day 35 postpartum, the glands were more abundant in the stratum spongiosum, along with increased peri glandular deposition of collagen (Figure 3D). In some samples, this analysis was limited due to incomplete epithelial preservation.

### 3.3. Characterization of Innate Immunity During the Postpartum Period

**Figure 4:**
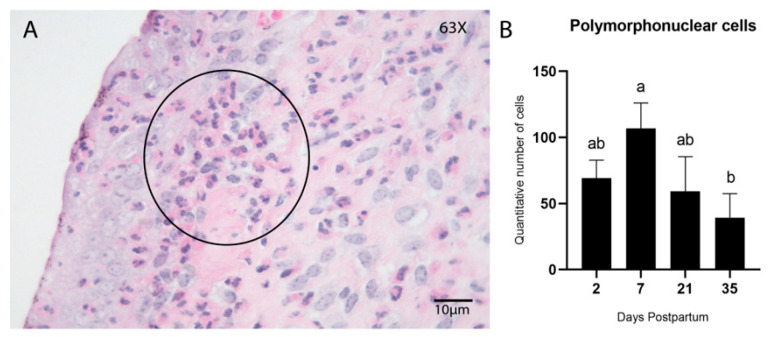
Infiltration of PMN cells in the endometrial stroma during postpartum. Hematoxylin and Eosin (HE) (**A**) Endometrial biopsy of bovine uterus. Representative image of the infiltration of PMN inflammatory cells in the endometrial stroma during the postpartum involution process (Day 7); a cluster of PMN cells is noticeable below the epithelium (encircled). The images were obtained using Aperio ImageScope x64 12.4.6 software (Leica Biosystems), magnification 630×, scale 10 µm. (**B**) Quantitative analysis of PMN cells showed a peak of increased infiltration on Day 7 postpartum, which gradually decreased by Day 35. (*p* < 0.05; Tukey–Kramer HSD test). Different letters are statistically significant **(***p* < 0.05). Results are presented as mean ± standard error of the mean (S.E.M.).

**Figure 5:**
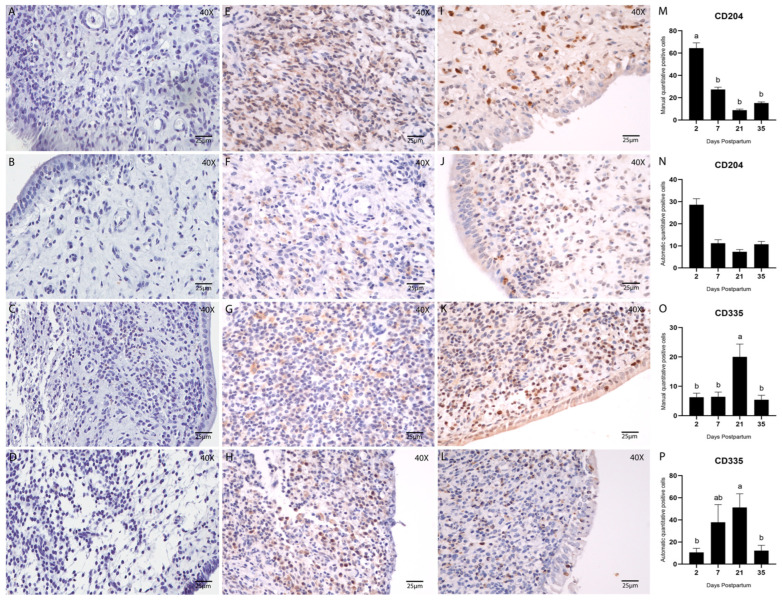
Immunostaining and quantification of Macrophage cells using the M2 macrophages CD204 (1:100) and NK cells using CD335 (1:200). Counterstained with Hematoxylin. (**A**–**D**) Negative controls, stained with hematoxylin only, are the same for all antibodies and were used to confirm tissue morphology preservation. (**E**–**H**) Endometrial biopsies of bovine uterus. Immunostaining for CD204 on Days 2, 7, 21, and 35 postpartum, respectively. (**I**–**L**) Endometrial biopsies of bovine uterus. Immunostaining for CD335 on Days 2, 7, 21, and 35 postpartum, respectively. The images were obtained using Aperio ImageScope x64 12.4.6 software (Leica Biosystems), magnification 400×, scale 25 µm. (**M**,**N**) Manual (**M**) and automatic (**N**) counting of CD204-positive cells. (**O**,**P**) Manual (**O**) and automatic (**P**) counting of CD335-positive cells. Different letters are statistically significant (*p* < 0.05). The absence of letters denotes no significant difference among groups. Results are presented as mean ± standard error of the mean (S.E.M.).

Early postpartum days were characterized by acute inflammation, marked by elevated infiltration of innate immune cells such as polymorphonuclear (PMN) cells, macrophages, and natural killer (NK) cells. PMN cells were evaluated in hematoxylin and eosin-stained sections (Figure 4A), whereas immunohistochemical detection of CD204 (Figure 5E–H) and CD335 (Figure 5I–L) was used to identify macrophages (M2 phenotype) and NK cells, respectively.

Quantification of macrophages and NK cells was performed using both manual counting and automated analysis in QuPath to evaluate consistency and correlation between the two methodologies. A significant difference in PMN cell counts was observed among postpartum time points, with the highest concentration on Day 7 (*p* = 0.03; Tukey–Kramer HSD test; Figure 4B). The number of PMN cells decreased progressively and was lowest on Day 35.

Macrophage quantification by CD204 immunostaining showed a significantly higher cell count on Day 2 postpartum in the manual analysis (*p* < 0.05; Tukey–Kramer HSD test; Figure 5M). However, no statistically significant difference was detected using the automated method (Figure 5N).

**Figure 6:**
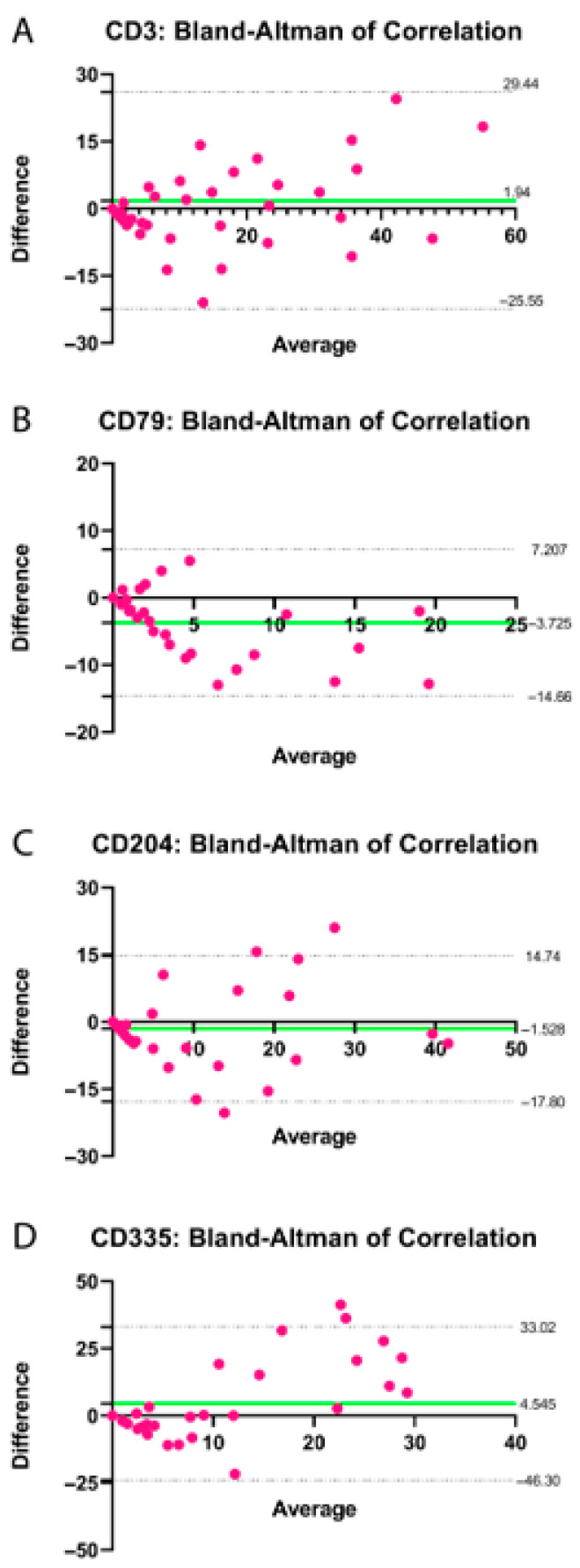
Bland–Altman plot showing the differences between manual and automated quantification of positively marked cells in the bovine endometrium for CD3 (**A**), CD79 (**B**), CD204 (**C**) and CD335 (**D**) during the postpartum period, plotted against the average of the two methods (pink dots). The bias is indicated by the distance between the zero-difference line (*X*-axis) and the solid green line. The limits of agreement are represented by the dashed lines parallel to the *X*-axis. Numerical values for the bias and limits of agreement are displayed adjacent to their respective lines.

NK cell quantification (CD335; 1:200) revealed a significant increase on Day 21 postpartum in both manual and automated analyses (*p* < 0.05; Tukey–Kramer HSD test; Figure 5O,P). Overall, the correlation between manual and automated quantification methods was considered satisfactory, as shown in Table 4 and Figure 6.

### 3.4. Characterization of Acquired Immunity During the Postpartum Period: Stromal Presence of B and T Lymphocytes

**Figure 7:**
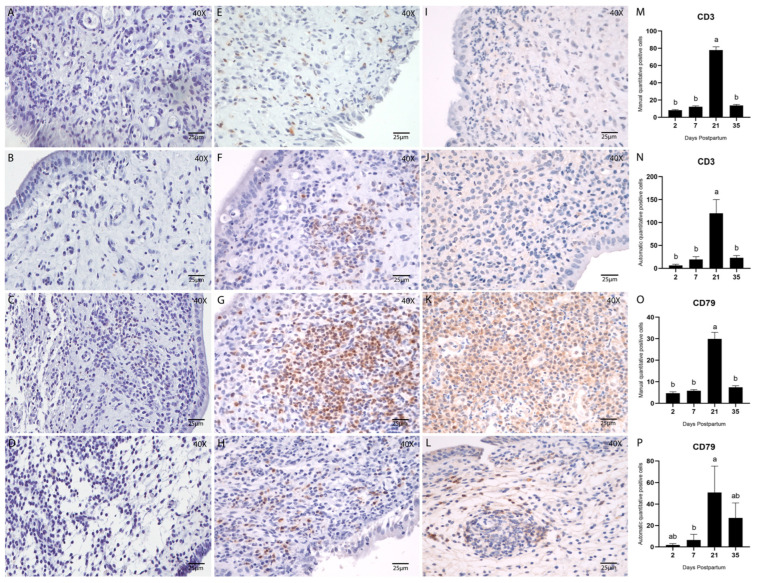
Endometrial biopsies of bovine uterus. Immunostaining and quantification of T cells using CD3 (1:100) and B-cells using CD79 (1:100) and counterstained with Hematoxylin. (**A**–**D**) Negative controls, stained with Hematoxylin only, are the same for all antibodies and were used to confirm tissue morphology preservation. (**E**–**H**) Endometrial biopsies of bovine uterus. Immunostaining for CD3 on Days 2, 7, 21, and 35 postpartum, respectively. (**I**–**L**) Endometrial biopsies of bovine uterus. Immunostaining for CD79 on Days 2, 7, 21, and 35 postpartum, respectively. The images were obtained using Aperio ImageScope x64 12.4.6 software (Leica Biosystems), magnification 400x, scale 25 µm. (**M**,**N**) Manual (**M**) and automatic (**N**) counting of CD3-positive cells. (**O**,**P**) Manual (**O**) and automatic (**P**) counting of CD79-positive cells. Different letters are statistically significant (*p* < 0.05). Results are presented as mean ± standard error of the mean (S.E.M.).

The presence and distribution of adaptive immune cells during uterine involution were assessed by immunohistochemical staining for T lymphocytes (CD3) (Figure 7E–H) and B lymphocytes (CD79) (Figure 7I–L). Quantification of both cell types was performed manually and via automated analysis using QuPath, as previously described.

T and B cells were consistently detected throughout the postpartum period. Quantitative analysis of B lymphocytes (CD79) yielded comparable results between manual and automated methods (Figure 7O,P). Both approaches demonstrated a significantly higher number of B cells on Day 21 postpartum (*p* < 0.05; Tukey–Kramer HSD test).

**Figure 8:**
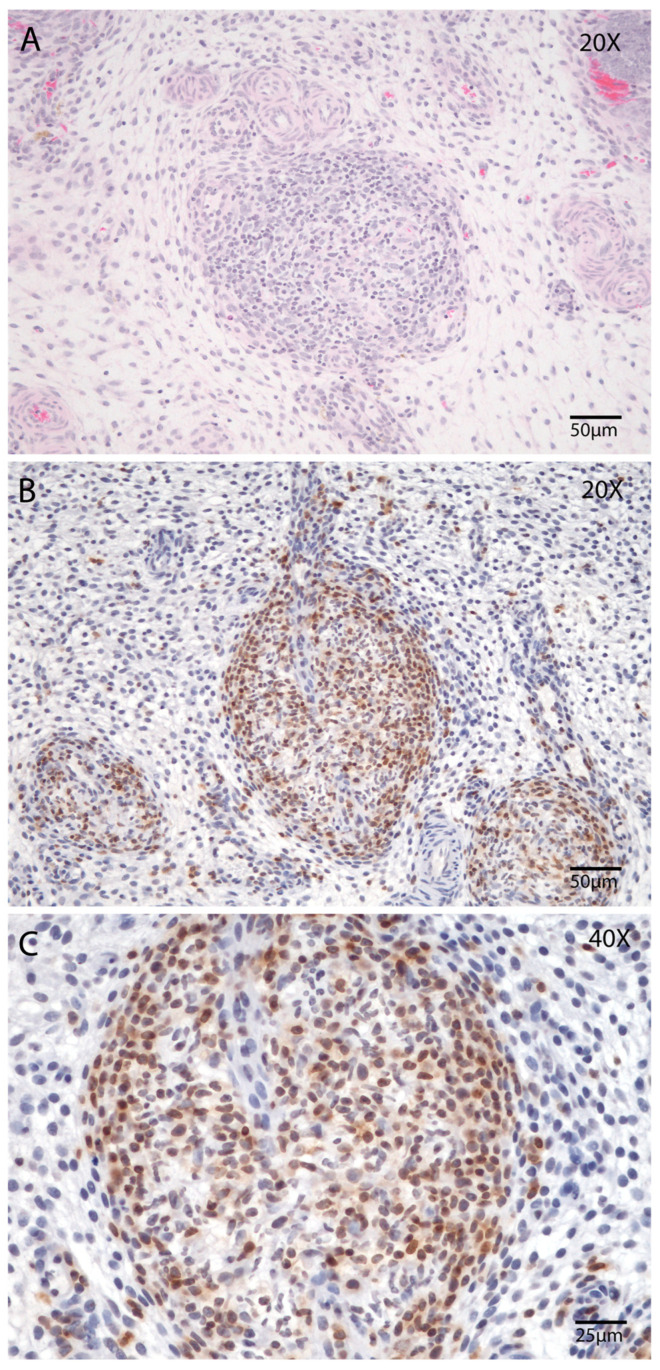
Presence of lymphoid nodules in the endometrial stroma during postpartum Day 21. (**A**,**B**) Endometrial biopsy of bovine uterus. (**A**) Lymphoid nodule in the endometrial stroma, Hematoxylin and Eosin (HE), magnification 200×, scale 50 µm. (**B**) Lymphoid nodules in the endometrial stroma immunostaining (counterstained with Hematoxylin), magnification 200×, scale 50 µm and (**C**) magnification 400×, scale 25 µm Immunostaining for T cells using the CD3 marker (1:100). The images were obtained using Aperio ImageScope x64 12.4.6 software (Leica Biosystems).

Similarly, the abundance of T lymphocytes (CD3) peaked on Day 21 postpartum. This was evident in both manual (*p* < 0.05; Tukey–Kramer HSD test; Figure 7M) and automated counts (*p* < 0.05; Tukey’s multiple comparison test; Figure 7N). Notably, at this point, the density of lymphocytes was sufficient to form discrete lymphocytic foci within the stroma (Figure 8A–C), a phenomenon rarely observed at other stages of involution in this study.

## 4. Discussion

Over the last few decades, numerous authors have objectively studied the physiological process of postpartum uterine involution in cows, using macroscopic, microscopic, and molecular analyses [6,11,25,26,27,28,29,30,31]. However, the available histological examinations of postpartum uterine involution in cows, conducted through endometrial biopsies, are based on tabulated data, long intervals between sample collections, and limited application of immunomarkers.

To support previous findings and expand knowledge on postpartum inflammation in healthy cows, our research group collected endometrial biopsies at four distinct time points, increasing the window during early postpartum. We subsequently evaluated morphological changes and the inflammatory cell profile in the endometrium using histology and immunohistochemistry. Recognized as a highly specific diagnostic method, endometrial biopsy is an invasive procedure that, although rarely, may potentially impair reproductive performance due to uterine injury [8]. Considering the reproductive safety of the animals involved in this study, and the frequent need for biopsy collection, we opted to use different animals to avoid compromising reproductive performance, ensuring ethical and methodological rigor.

Taken together, these biopsies provided a robust basis for comprehensive analysis, with 95% permitting a complete evaluation of the characteristics of interest, in line with findings from previous studies [25]. It is well recognized that the timing of biopsy collection, particularly during the postpartum period, can influence sample quality and affect both epithelial integrity and classification [25]. Importantly, our data did not show any adverse effects on epithelial integrity across the sampling days. The presence of intact epithelium in 92.98% of the collected biopsies enabled successful epithelial characterization and classification. The epithelium was consistently classified as columnar in all samples.

Although uterine involution is typically associated with marked tissue remodeling and inflammation, including epithelial disruption and hemorrhage, especially in the early postpartum period, the degree of these changes can vary according to the uterine region evaluated. The caruncular areas, which undergo placental detachment, are usually more affected, showing necrosis, sloughing of epithelium, and vascular lesions [25,32]. Conversely, the intercaruncular regions tend to exhibit less severe histological changes, with more rapid epithelial regeneration [33]. This may explain the relatively preserved epithelium and limited bleeding observed in our Day 2 and Day 7 samples. Regarding this, samples that appeared to originate from caruncular regions were excluded from the analysis to ensure consistency in the evaluation of intercaruncular tissue remodeling and immune response. This approach aimed to minimize variability due to regional differences in uterine histology during the early postpartum period.

No significant alterations were detected in the cellular composition of the stratum compactum and stratum spongiosum during the involution process. As previously reported [34,35], the compactum layer exhibited a higher density of stromal cells, while PMNs, and mononuclear inflammatory cells increased or decreased according to different postpartum Days. Additionally, an abundance of blood vessels in the stratum compactum was observed, with increased congestion in the early postpartum period and a larger vessel diameter on Day 35. Although numerous blood vessels, edema, and hemorrhage were noted in the stratum compactum, these factors potentially contribute to an increased influx of inflammatory cells—especially PMNs—during the uterine involution period [27,34], our findings showed no significant correlation between hemorrhage and PMN counts.

Notably, on Day 35 postpartum, we observed a higher number of endometrial glands. This increase may reflect a return to cyclicity and restoration of glandular function, aligning with previous studies [25]. The endometrial glands play a crucial role in fertility, and endometrial disorders have been shown to impede their development during the early postpartum period [36], which may explain the lower number of endometrial glands observed on day 7 compared to day 35. Moreover, the intense immune activity and tissue remodeling that occur in the early postpartum period may also contribute to the reduced number of endometrial glands on Day 7, as these processes can transiently disrupt epithelial integrity and delay glandular regeneration. Research indicates that cows in the luteal phase of the estrous cycle exhibit a greater concentration of endometrial glands [37]. It is established that between postpartum days 46 and 54, the glands regain their normal condition, as evidenced by the external and internal diameters and their distribution in the endometrial tissue [38]. Our observations align with studies proposing that postpartum uterine involution is complete by day 40 or later [38]. However, the return to cyclicity of the animals included in this study was not assessed, which may have influenced the results and warrant consideration in future investigations.

Histological analyses in sheep have shown a reduction in the extracellular space within the endometrial stroma and a concurrent increase in extracellular matrix deposition during uterine involution, suggesting active tissue remodeling in both caruncular and intercaruncular regions of the uterine wall [33]. Our evaluation of endometrial biopsies did not reveal significant differences in collagen fiber density, peri glandular fibrosis, or the stratum compactum. However, our results still suggest degradation and remodeling of the extracellular matrix, as we observed higher collagen levels on Day 2 postpartum, followed by a gradual decrease and reorganization. Additionally, a higher concentration of subepithelial collagen fibers was noted on Day 21 postpartum, potentially indicating proper remodeling of the stratum compactum by this time. This may indicate that matrix remodeling and tissue repair processes are particularly active during this period. While existing studies suggest rapid digestion of collagen postpartum, primarily through macrophage activity [39], our histological distinctions between the early and late stages of uterine involution were not as pronounced. However, additional experiments are required to properly characterize the ongoing collagen degradation during involution, perhaps through the evaluation of different active metalloproteinases associated with collagen degradation in the uterus [40].

As the first line of defense against microorganisms in the uterine lumen, epithelial cells play a crucial role, with neutrophils being the primary and most significant phagocytic cells recruited into this environment [11]. Distinct immune and inflammatory processes occur at different layers of the endometrium. For instance, neutrophils are almost exclusively located on the endometrial surface, while deeper layers such as the stroma exhibit different immune cell profiles. There is substantial evidence that detecting more than 5% neutrophils in cytology smears is associated with reduced fertility, indicating active endometrial inflammation [41]. The prevalence of biopsies showing PMN cells as the predominant inflammatory cell type in the early postpartum period may indicate an acute tissue reaction [25].

Phagocytic cells, including neutrophils and macrophages, eliminate invading bacteria through coordinated processes of adhesion, attachment, ingestion, and digestion [32]. In the present study, we consider PMN cells, macrophages, and NK cells as key regulators of the acute immune response during the early postpartum period. Notably, an increase in PMN cell numbers on Days 2 and 7 postpartum, as demonstrated by Bonnet et al. [25].

Extracellular matrix (ECM) remodeling is essential for postpartum uterine involution in dairy cows, involving a dynamic interplay between different macrophage populations [42]. Macrophage infiltration was notably higher on Day 2 postpartum. During postpartum, there is an increase in pro-inflammatory M1 macrophages, followed by a transition to reparative M2 macrophages. M2 macrophages promote type I collagen synthesis, contributing to the progressive deposition of this protein in the endometrial tissue. Additionally, the scavenger receptor CD204 facilitates the clearance of apoptotic cells, supporting tissue repair and stromal cell expansion [43]. These combined factors explain the observed increase in CD204 expression on Day 2 postpartum.

Reduced peripheral T and B cells populations have been observed in cows with delayed uterine involution, a condition associated with increased risk of uterine disease. Some findings suggest that prepartum changes in CD3 T cell proportions may play a role in impaired uterine involution and heightened susceptibility to uterine disease [44,45]. Authors showed the subepithelial uterine stroma exhibits a notably higher density of CD4^+^ T cells, B cells, CD14^+^ macrophages, and mast cells relative to other regions of the endometrium and myometrium [46]. Our findings reveal a significant increase in lymphocyte infiltration within the stratum compactum at Day 21 postpartum involution, with occasional formation of lymphocytic aggregates. Lymphocytic foci in the uterine endometrium, often linked to previous postpartum uterine disease, have been associated with reduced placental weight during early pregnancy [47]. These structures are thought to arise from chronic inflammation following uterine infection and may persist for prolonged periods [48]. It is hypothesized that neutrophil infiltration during the early postpartum period, as showed on Day 7, initiates this process, leading to the formation of lymphocytic aggregates that can modify endometrial function and compromise its capacity to support placental development [47]. Alternatively, these foci may serve as inductive sites for local T and B cell responses, contributing to immune protection against secondary uterine infections, while also acting as histological markers of prior endometrial injury [47,49]. These observations corroborate prior studies reporting analogous immune cell dynamics, which were further supported by elevated CD45 gene expression [17,25].

Day 35 postpartum is commonly utilized for the diagnosis of endometritis through the detection of neutrophils via endometrial cytobrush sampling. However, our findings raise the hypothesis that endometritis might be detectable at an earlier stage, given the reduced neutrophil presence observed in biopsies collected on Day 21. Furthermore, they may participate in the immunological protection against uterine infections, by way of cytotoxicity as well as their cytokine-producing abilities [50]. Notably, the inflammatory profile at this earlier time point reveals a significant shift characterized by increased lymphocytic infiltration and elevated natural killer (NK) cell populations. By Day 35 the lymphocyte-dominant inflammation subsequently regresses, suggesting a dynamic and temporally regulated immune response during postpartum endometrial involution. The precise mechanisms underlying this observation remain unclear, raising important questions regarding the signaling pathways that drive this peak inflammatory response and its potential effects on the uterine microbiota and endometrial health. The immunophenotype of the receptive endometrium is characterized by the accumulation of NK cells, which actively proliferate, mature, differentiate, and prepare to support embryo implantation, increasing their proportion during the implantation window. Endometrial receptivity is largely influenced by this immunophenotype, with NK cells playing a central role [51,52]. The observed increase in CD335 expression on Day 21 postpartum may result from this cyclic modulation of NK cell populations. Nonetheless, further investigations incorporating both biopsy and cytological analyses are necessary to validate these hypotheses.

In this study, we compared manual quantitative counts with automated quantification using QuPath© software. The protocol efficiently identifies positively stained immune cells, allowing assessment of immune cell proportion and density across tissue regions. Its ability to analyze entire tissue sections rapidly provides a major advantage over manual counting [53]. Our results demonstrated a strong correlation between the two methods. Understanding the number and types of inflammatory cells at each stage of uterine involution in cows holds the potential to serve as a diagnostic tool, enabling the anticipation of animals susceptible to develop uterine diseases. However, discrepancies may occur due to subjectivity in manual counts and area coverage or parameter settings in the automated approach.

Dysregulation of this inflammatory process may predispose cows to uterine pathologies and subfertility. Characterizing the quantity and phenotype of inflammatory cells at distinct stages could aid in early detection of at-risk animals, potentially improving reproductive outcomes in dairy herds. Differences in physiological responses between primiparous and multiparous cows during the transition period have been well documented and may significantly influence immune, metabolic, and oxidative parameters [54]. These parity-related differences may affect uterine recovery dynamics and should be carefully considered in studies investigating postpartum physiology. While the present study was restricted to multiparous animals, future investigations that incorporate parity as a stratifying variable are warranted to provide a more comprehensive understanding of its role in uterine involution and postpartum adaptation.

## 5. Conclusions

In conclusion, despite being an invasive procedure, endometrial biopsy remains an invaluable tool for comprehensive assessment of endometrial status. Although histological and immunohistochemical analyses may not be feasible for routine veterinary diagnostics, they are essential for advancing research into postpartum endometrial involution and uterine health. The integration of morphological findings with the quantification of inflammatory and molecular markers provides valuable insights into the mechanisms underlying subclinical endometritis. The morphological findings here reinforce the hypothesis that immune regulation during uterine involution follows a dynamic timeline, with potential diagnostic value for subclinical conditions.

Our results showed a dynamic modulation of the inflammatory profile throughout uterine involution. Notably, a shift from neutrophilic to lymphocytic cell infiltration in the endometrial stroma was observed around Day 21 postpartum, indicating a potential marker for deviations from physiological involution. The persistence of polymorphonuclear neutrophils beyond this period may signal a prolonged inflammatory response. Additionally, the increased presence of NK cells at Day 21 warrants further investigation into their role in the return to cyclicity. These findings support the development of reference standards to improve the early detection and management of subclinical endometritis. Future studies should also explore the contribution of the uterine microbiome and immune tolerance mechanisms during postpartum recovery.

## Figures and Tables

**Table 1:** Description of the criteria used for the morphological characterization of uterine biopsies.

Area	Criteria	Subjective Assessment	Quantitative Assessment
Biopsy	Readability	Unreadable, readable, good, very good	Number of areas where epithelium could be measure
Epithelium	Height	Columnar, cuboidal flattened	Intact epithelium measured at 400×
	Inflammatory cells	Mononuclear, PMN infiltrating the epithelium	Number (400×)
	Edema		Grade (0–4) (200× to 400×)
	Hemorrhage		Grade (0–4) (200× to 400×)
Stratum compactum	Inflammatory cells	Mononuclear, PMN noted in the stratum compactum	Number (400×)
	Lymphocytic foci		Number in biopsy (100×)
	Edema		Grade (0–4) (200× to 400×)
	Vasodilation		Grade (0–4) (200× to 400×)
	Hemorrhage		Grade (0–4) (200× to 400×)
Stratum spongiosum	Lymphocytic foci		Number in biopsy (100×)
Endometrial Glands	Concentration	None, scarce, moderate, heavy	Number (100×)
	Type	Straight, coiled	
	Size	Dilated glands, present/absent	Inner and outer gland diameter
	Fibrosis	Present/absent	Counts of layers of fibroblasts around glands
	Inflammatory cells	Mononuclear, PMN noted around glands	Number (400×)

**Table 2:** List of primary and secondary antibodies selected for immunohistochemistry.

Name of Antibody	Manufacturer (Cat. No.)	Type	Dilution
CD204	Cosmo Bio LTD; Tokio, Japan (KAL-KT022)	MoM	1:100
CD79	Novus Biologicals; Centennial, CO, USA (NB-1006434 7)	MoM	1:100
CD3	Biorad; Kidlington, Oxfordshire, United Kingdom (MCA1477T)	RatM	1:100
NKp46 (CD335)	Thermo Fisher; Waltham, MA, USA (PA5-102860)	RbP	1:200
Goat Anti-Rat IgG (H + L)	Vector Laboratories; Newark, CA, USA (VECTBA9400)	Rat	1:200
Goat Anti-Mouse IgG (H + L)	Vector Laboratories; Newark, CA, USA (VECTBA9200)	Mo	1:200
Goat Anti-Rabbit IgG	Vector Laboratories; Newark, CA, USA (PK-4001)	Rb	1:200

Mo: Mouse; MoM: Mouse monoclonal; Rat: Rat; RatM: Rat monoclonal; Rb: Rabbit; RbP: Rabbit polyclonal.

**Table 3:** Summary of the number and categorization of endometrial glands in endometrial biopsies.

Postpartum	Concentration		Type	Size	Inflammation (Around Glands)	Fibrosis (Around Glands)
Day 2 (*n =* 11)	None	20.0%	Straight	Dilated	Present	55%
	Scarce	60.0%				
	Heavy	20.0%				
Day 7 (*n =* 15)	Scarce	100%	Straight	Dilated	Present	83%
Day 21 (*n =* 13)	Scarce	18.1%	Coiled	Dilated	Present	70%
	Moderate	45.4%				
	Heavy	36.3%				
Day 35 (*n =* 11)	Moderate	20.0%	Coiled	Dilated	Present	33%
	Heavy	80.0%				

**Table 4:** Descriptive statistical analysis of the correlation between manual and automated quantification of positively marked cells in the bovine endometrium during postpartum period.

	Day PPT	CD3	CD79	CD204	CD335
Correlation (r-value)	2	0.4468	0.7065	0.7367	−0.1423
Spearman’s p		0.8151	0.5411	0.7792	0.3537
*p*-value		0.0022 *	0.0856	0.0079 *	0.286
Correlation (r-value)	7	0.6577	0.4313	0.8231	−0.1581
Spearman’s p		0.8248	0.4276	0.8831	0.467
*p*-value		0.0002 *	0.1118	0.0001 *	0.0792
Correlation (r-value)	21	0.6909	0.5164	0.3852	−0.1212
Spearman’s p		0.7198	0.7652	0.5712	0.0414
*p*-value		0.0055 *	0.0023 *	0.0524	0.8932
Correlation (r-value)	35	0.6835	0.0808	0.1821	−0.1527
Spearman’s p		0.492	0.4361	0.4977	−0.1918
*p*-value		0.1242	0.18	0.1193	0.5721
Correlation (r-value)	all Days	0.8232	0.51	0.7059	−0.0375
Spearman’s p		0.8544	0.6033	0.8150	0.2758
*p*-value (*p* < 0.05)		<0.0001 *	<0.0001 *	<0.0001 *	0.0526 *

* Significant *p*-value (*p* < 0.05).

## Data Availability

Data can be shared by authors upon reasonable request.

## Supplementary Materials

The following supporting information can be downloaded at https://www.mdpi.com/article/10.3390/ani15172520/s1: Figure S1: Comparative image of type and size of endometrial glands. Figure S2: Comparative image of assessing Immunostaining for T cells using the CD3 marker (1:100) using QuPath© software.
