# Supplementary figures and images for: Postpartum Uterine Involution in Cows: Quantitative Assessment of Structural Remodeling and Immune Cell Infiltration
Source: Animals (Basel). 2025 Aug 27;15(17):2520. doi: 10.3390/ani15172520 (PMC12427534; doi:10.3390/ani15172520)

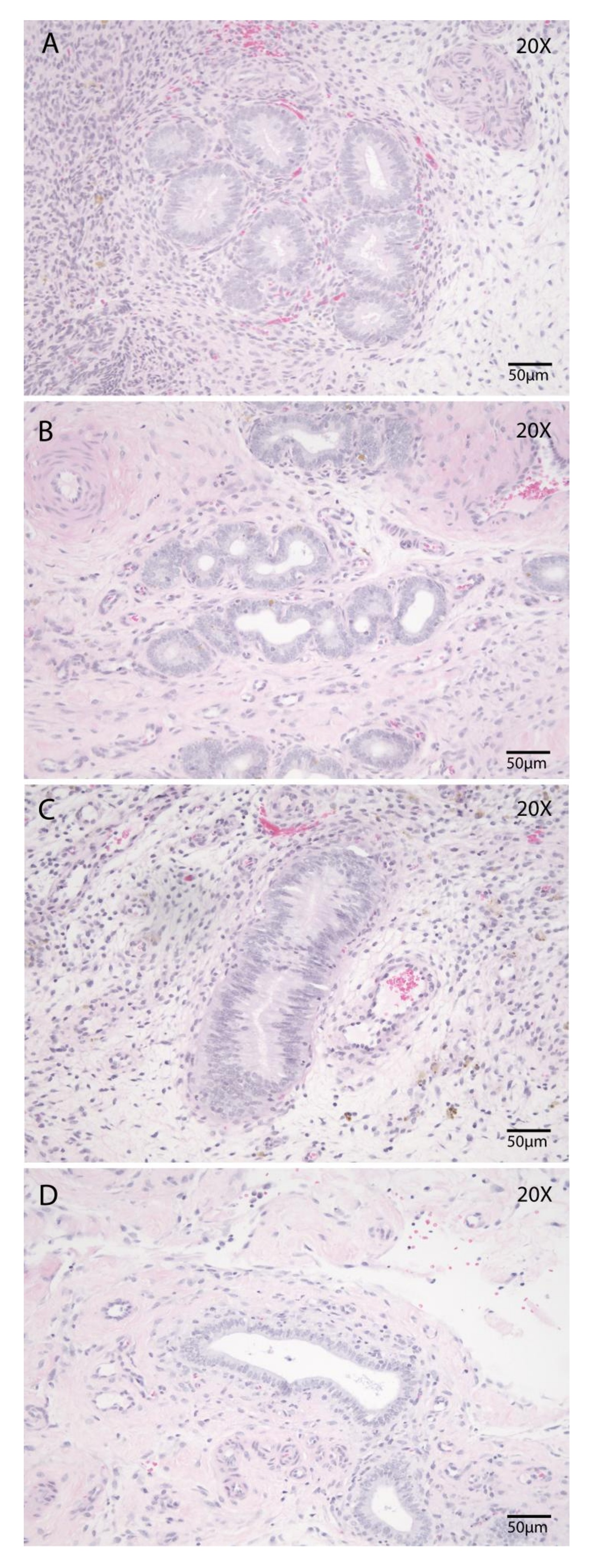

Supplement: Supplementary file 1 [file animals-15-02520-s001.zip › Figure S1.jpg]
